# Crystal structure of bis­[2,5-bis­(pyridin-2-yl)-1,3,4-thia­diazole-κ^2^ *N* ^2^,*N* ^3^]bis­(thio­cyanato-κ*S*)copper(II)

**DOI:** 10.1107/S2056989016011713

**Published:** 2016-07-22

**Authors:** Abdelhakim Laachir, Fouad Bentiss, Salaheddine Guesmi, Mohamed Saadi, Lahcen El Ammari

**Affiliations:** a Laboratoire de Chimie de Coordination et d’Analytique (LCCA), Faculté des Sciences, Université Chouaib Doukkali, BP 20, M-24000 El Jadida, Morocco; b Laboratoire de Catalyse et de Corrosion de Matériaux (LCCM), Faculté des Sciences, Université Chouaib Doukkali, BP 20, M-24000 El Jadida, Morocco; c Laboratoire de Chimie du Solide Appliquée, Faculty of Sciences, Mohammed V University in Rabat, Avenue Ibn Battouta, BP 1014, Rabat, Morocco

**Keywords:** crystal structure, copper complex, 2,5-bis­(pyridin-2-yl)-1,3,4-thia­diazole, thio­cyanate ligand

## Abstract

The mononuclear title complex, [Cu(SCN)_2_(C_12_H_8_N_4_S)_2_], was obtained by the reaction of 2,5-bis­(pyridin-2-yl)-1,3,4-thia­diazole and potassium thio­cyanate with copper(II) chloride dihydrate. The copper cation lies on an inversion centre and displays an elongated octa­hedral coordination geometry. The equatorial positions are occupied by the N atoms of two 2,5-bis­(pyridin-2-yl)-1,3,4-thia­diazole ligands, whereas the axial positions are occupied by the S atoms of two thio­cyanate anions. The thia­diazole and the pyridyl rings linked to the metal are approximately coplanar, with a maximum deviation from the mean plane of 0.190 (2) Å. The cohesion of the crystal structure is ensured by weak C—H⋯N hydrogen bonds and π–π inter­actions between parallel pyridyl rings of neighbouring mol­ecules [centroid-to-centroid distance = 3.663 (2) Å], leading to a three-dimensional network.

## Abstract 2

The structure of the title compound is similar to that of the related complexes [Co(C_12_H_8_N_4_S)_2_(N_3_)_2_] and [Ni(C_12_H_8_N_4_S)_2_(N_3_)_2_] in which the azide ion is substituted by the thio­cyanate group. The CuN_4_S_2_ octa­hedron is more distorted than the CoN_6_ and NiN_6_ octa­hedra.

## Chemical context   

The use of compounds containing a 1,3,4-thia­diazole moiety as part of ligand systems has gained considerable attention in recent years (Kadam Sushama *et al.*, 2016 ▸). Indeed, a 2,5-bis­(pyridin-2-yl)-1,3,4-thia­diazole (bptd) and its metal complexes have been extensively studied because of their potential applications in biology (Baghel *et al.*, 2014 ▸; Ahmed *et al.*, 2015 ▸; Zine *et al.*, 2016 ▸), magnetism (Bentiss *et al.*, 2004 ▸) and coordination chemistry (Bentiss *et al.*, 2002 ▸). An inter­esting feature of the metal-ligand chemistry of these compounds is that the complexes can be mononuclear (Bentiss *et al.*, 2011 ▸, 2012 ▸; Klingele *et al.*, 2010 ▸; Kaase & Klingele, 2014 ▸) or binuclear (Laachir *et al.*, 2013 ▸). 
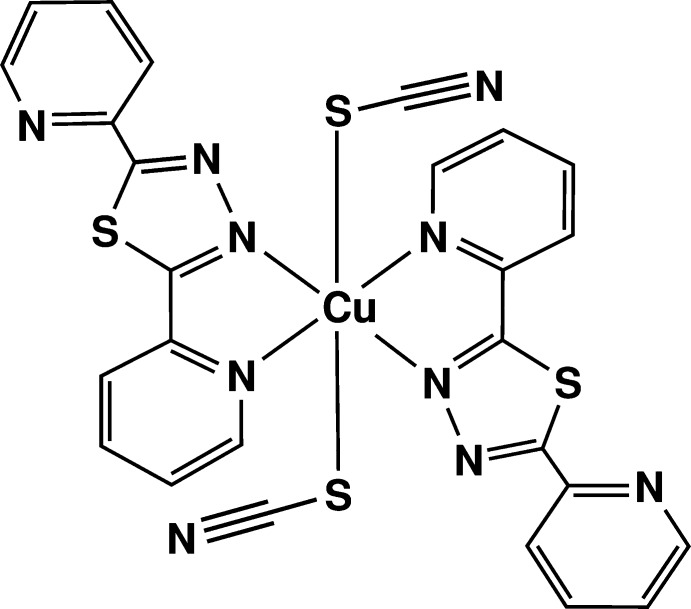


We have recently reported the synthesis and characterization of monomeric complexes of Ni^II^ and Co^II^ with bptd in the presence of the pseudohalide azide (Laachir *et al.*, 2015*a* ▸,*b* ▸). In this context, we report here the synthesis and crystal structure of a new Cu^II^ complex with bptd and thio­cyanate as co-ligands.

## Structural commentary   

**Figure 1:**
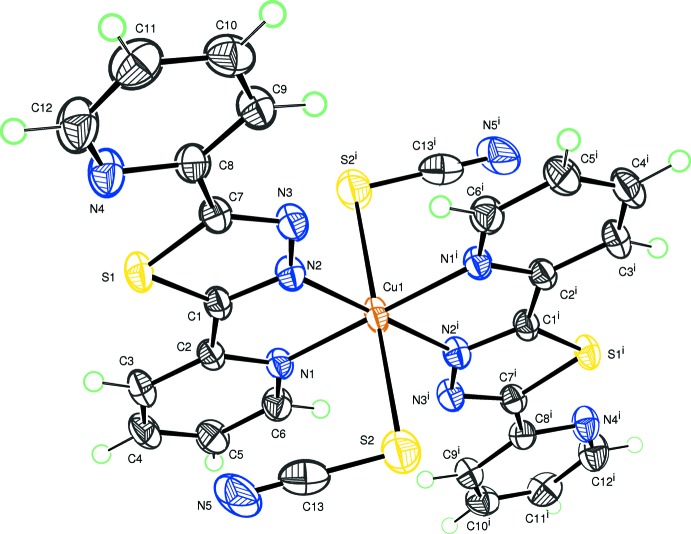
The mol­ecular structure of the title compound, with displacement ellipsoids drawn at the 50% probability level. H atoms are represented as small circles. [Symmetry code: (i) −*x* + 1, −*y* + 1, −*z* + 1.]

The title complex has crystallographically imposed inversion symmetry, the copper atom lying on the Wyckoff special position *2b* of the space group *P*2_1_/*c*. The elongated octa­hedral coordination polyhedron around the metal cation is provided by four nitro­gen atoms of pyridine and thia­diazole rings occupying the equatorial plane and by the sulfur atoms of two thio­cyanate anions at the apical positions (Fig. 1 ▸). The Cu—N distances are 2.0267 (16) and 2.0463 (15) Å, the Cu—S bond length is 2.8125 (7) Å. A bond-valence-sum calculation (Brown & Altermatt, 1985 ▸) for Cu gives the expected BVS value of 2.11 valence units. The conformation of the ligand is approximately planar, with a maximum deviation from the least-squares plane of 0.190 (2) Å for atom C12. The dihedral angles formed by the thia­diazole ring with the N1/C2–C6 and N4/C8–C12 pyridine rings are 1.94 (8) and 6.96 (5)°, respectively.

## Supra­molecular features   

**Figure 2:**
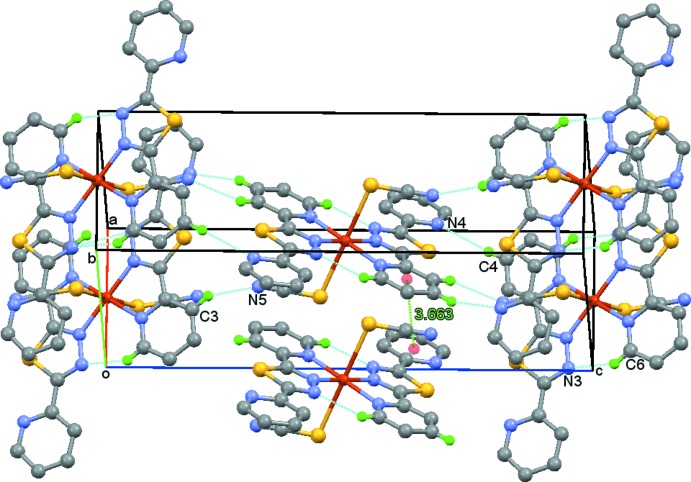
Crystal packing of the title compound, showing π–π inter­actions between pyridyl rings (green dashed lines) and inter­molecular hydrogen bonds (blue dashed lines).

In the crystal, the mol­ecules are linked by weak C—H⋯N hydrogen bonds (Table 1 ▸) and by π–π stacking inter­actions between the pyridyl rings of adjacent complex mol­ecules [inter­centroid distance = 3.663 (2) Å], forming a three-dimensional network (Fig. 2 ▸).

## Database survey   

The structure of the title compound is similar to that of the related complexes [Co(C_12_H_8_N_4_S)_2_(N_3_)_2_] (Laachir *et al.*, 2015*b* ▸) and [Ni(C_12_H_8_N_4_S)_2_(N_3_)_2_] (Laachir *et al.*, 2015*a* ▸), in which the azide ion is substituted by the thio­cyanate group. The CuN_4_S_2_ octa­hedron is more distorted than the NiN_6_ and CoN_6_ octa­hedra.

## Synthesis and crystallization   

2,5-Bis(pyridin-2-yl)-1,3,4-thia­diazole (bptd) was synthesized as described previously by Lebrini *et al.* (2005 ▸). A solution of bptd (24 mg, 0.1 mmol) in CH_3_CN (10 mL) was layered onto a solution of CuCl_2_·2H_2_O (17 mg, 0.1 mmol) and KSCN (20 mg, 0.2 mmol) in CH_3_CN/H_2_O (1:1 *v*/*v*, 10 mL) in a test tube. The solution was left for two months at room temperature to give X-ray quality brown block-shaped crystals. After filtration, the product was washed with cold EtOH and dried under vacuum. Crystals were washed with water and dried under vacuum (yield 60%; m.p. 538 K). Analysis calculated for C_26_H_16_N_10_S_4_Cu: C, 47.30; H, 2.44; N, 21.21 S, 19.42. Found: C, 47.06; H, 2.43; N, 21.03; S, 19.56.

## Refinement   

Crystal data, data collection and structure refinement details are summarized in Table 2 ▸. H atoms were located in a difference Fourier map and treated as riding, with C—H = 0.96 Å, and with *U*_iso_(H) = 1.2 *U*_eq_(C). One outlier (002) was omitted in the last cycles of refinement.

## Supplementary Material

Crystal structure: contains datablock(s) I. DOI: 10.1107/S2056989016011713/rz5192sup1.cif

Structure factors: contains datablock(s) I. DOI: 10.1107/S2056989016011713/rz5192Isup2.hkl

CCDC reference: 1494615

Additional supporting information: crystallographic information; 3D view; checkCIF report

## Figures and Tables

**Table 1:** Hydrogen-bond geometry (Å, °)

*D*—H⋯*A*	*D*—H	H⋯*A*	*D*⋯*A*	*D*—H⋯*A*
C3—H3⋯N5^i^	0.93	2.53	3.353 (3)	147
C6—H6⋯N3^ii^	0.93	2.35	3.143 (3)	142
C4—H4⋯N4^iii^	0.93	2.57	3.458 (3)	161

Symmetry codes: (i) 
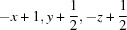
; (ii) 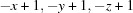; (iii) 
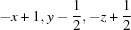
.

**Table 2:** Experimental details

Crystal data	
Chemical formula	[Cu(SCN)_2_(C_12_H_8_N_4_S)_2_]
*M*_r_	660.27
Crystal system, space group	Monoclinic, *P*2_1_/*c*
Temperature (K)	296
*a*, *b*, *c* (Å)	8.0205 (3), 7.8434 (3), 21.3454 (9)
β (°)	92.565 (2)
*V* (Å^3^)	1341.45 (9)
*Z*	2
Radiation type	Mo *K*α
μ (mm^−1^)	1.17
Crystal size (mm)	0.35 × 0.32 × 0.26

Data collection	
Diffractometer	Bruker X8 APEX
Absorption correction	Multi-scan (*SADABS*; Krause *et al.*, 2015 ▸)
*T*_min_, *T*_max_	0.604, 0.746
No. of measured, independent and observed [*I* > 2σ(*I*)] reflections	42199, 4089, 3155
*R*_int_	0.060
(sin θ/λ)_max_ (Å^−1^)	0.714

Refinement	
*R*[*F* ^2^ > 2σ(*F* ^2^)], *wR*(*F* ^2^), *S*	0.036, 0.097, 1.04
No. of reflections	4089
No. of parameters	187
H-atom treatment	H-atom parameters constrained
Δρ_max_, Δρ_min_ (e Å^−3^)	0.56, −0.51

Computer programs: *APEX2* and *SAINT* (Bruker, 2009 ▸), *SHELXT* (Sheldrick, 2015*a* ▸), *SHELXL2014* (Sheldrick, 2015*b* ▸), *ORTEPIII* (Burnett & Johnson, 1996 ▸), *ORTEP-3 for Windows* (Farrugia, 2012 ▸), *Mercury* (Macrae *et al.*, 2008 ▸) and *publCIF* (Westrip, 2010 ▸).

## supplementary crystallographic information

### Crystal data

**Table:**

[Cu(NCS)_2_(C_12_H_8_N_4_S)_2_]	*D*_x_ = 1.635 Mg m^−^^3^
*M**_r_* = 660.27	Melting point: 538 K
Monoclinic, *P*2_1_/*c*	Mo *K*α radiation, λ = 0.71073 Å
*a* = 8.0205 (3) Å	Cell parameters from 4089 reflections
*b* = 7.8434 (3) Å	θ = 2.5–30.5°
*c* = 21.3454 (9) Å	µ = 1.17 mm^−^^1^
β = 92.565 (2)°	*T* = 296 K
*V* = 1341.45 (9) Å^3^	Block, brown
*Z* = 2	0.35 × 0.32 × 0.26 mm
*F*(000) = 670	

### Data collection

**Table:**

Bruker X8 APEX diffractometer	4089 independent reflections
Radiation source: fine-focus sealed tube	3155 reflections with *I* > 2σ(*I*)
Graphite monochromator	*R*_int_ = 0.060
φ and ω scans	θ_max_ = 30.5°, θ_min_ = 2.5°
Absorption correction: multi-scan (SADABS; Krause *et al.*, 2015)	*h* = −11→11
*T*_min_ = 0.604, *T*_max_ = 0.746	*k* = −11→11
42199 measured reflections	*l* = −27→30

### Refinement

**Table:**

Refinement on *F*^2^	0 restraints
Least-squares matrix: full	Hydrogen site location: inferred from neighbouring sites
*R*[*F*^2^ > 2σ(*F*^2^)] = 0.036	H-atom parameters constrained
*wR*(*F*^2^) = 0.097	*w* = 1/[σ^2^(*F*_o_^2^) + (0.0418*P*)^2^ + 0.834*P*] where *P* = (*F*_o_^2^ + 2*F*_c_^2^)/3
*S* = 1.04	(Δ/σ)_max_ < 0.001
4089 reflections	Δρ_max_ = 0.56 e Å^−^^3^
187 parameters	Δρ_min_ = −0.51 e Å^−^^3^

### Special details

**Table:**

Geometry. All esds (except the esd in the dihedral angle between two l.s. planes) are estimated using the full covariance matrix. The cell esds are taken into account individually in the estimation of esds in distances, angles and torsion angles; correlations between esds in cell parameters are only used when they are defined by crystal symmetry. An approximate (isotropic) treatment of cell esds is used for estimating esds involving l.s. planes.

### Fractional atomic coordinates and isotropic or equivalent isotropic displacement parameters (Å^2^)

**Table:**

	*x*	*y*	*z*	*U*_iso_*/*U*_eq_	
C1	0.4345 (2)	0.6803 (2)	0.38733 (8)	0.0240 (4)	
C2	0.5958 (2)	0.6037 (2)	0.37724 (8)	0.0240 (4)	
C3	0.6786 (3)	0.6181 (3)	0.32209 (9)	0.0327 (4)	
H3	0.6314	0.6778	0.2881	0.039*	
C4	0.8333 (3)	0.5416 (3)	0.31863 (10)	0.0372 (5)	
H4	0.8909	0.5469	0.2818	0.045*	
C5	0.9012 (3)	0.4572 (3)	0.37055 (11)	0.0366 (5)	
H5	1.0065	0.4079	0.3696	0.044*	
C6	0.8102 (3)	0.4473 (3)	0.42395 (10)	0.0320 (4)	
H6	0.8563	0.3896	0.4586	0.038*	
C7	0.1712 (2)	0.8133 (2)	0.39355 (9)	0.0251 (4)	
C8	0.0124 (2)	0.9022 (3)	0.38144 (9)	0.0270 (4)	
C9	−0.1027 (3)	0.9212 (3)	0.42773 (10)	0.0322 (4)	
H9	−0.0804	0.8796	0.4680	0.039*	
C10	−0.2511 (3)	1.0036 (3)	0.41222 (12)	0.0387 (5)	
H10	−0.3306	1.0198	0.4421	0.046*	
C11	−0.2792 (3)	1.0615 (3)	0.35150 (12)	0.0430 (5)	
H11	−0.3782	1.1168	0.3396	0.052*	
C12	−0.1570 (3)	1.0356 (3)	0.30870 (12)	0.0439 (6)	
H12	−0.1772	1.0747	0.2679	0.053*	
C13	0.3302 (3)	0.2765 (3)	0.36867 (13)	0.0423 (5)	
N1	0.65894 (19)	0.5170 (2)	0.42789 (7)	0.0246 (3)	
N2	0.36607 (19)	0.6566 (2)	0.44138 (7)	0.0258 (3)	
N3	0.2129 (2)	0.7323 (2)	0.44510 (7)	0.0282 (3)	
N4	−0.0124 (2)	0.9581 (2)	0.32252 (8)	0.0354 (4)	
N5	0.3255 (3)	0.3117 (3)	0.31799 (10)	0.0568 (6)	
Cu1	0.5000	0.5000	0.5000	0.02713 (10)	
S1	0.31614 (6)	0.80256 (7)	0.33603 (2)	0.02941 (12)	
S2	0.33021 (9)	0.22277 (9)	0.44310 (3)	0.04809 (17)	

### Atomic displacement parameters (Å^2^)

**Table:**

	*U*^11^	*U*^22^	*U*^33^	*U*^12^	*U*^13^	*U*^23^
C1	0.0269 (9)	0.0275 (9)	0.0174 (8)	−0.0018 (7)	0.0005 (6)	0.0016 (7)
C2	0.0256 (8)	0.0274 (9)	0.0191 (8)	−0.0026 (7)	0.0017 (6)	−0.0008 (7)
C3	0.0359 (10)	0.0429 (12)	0.0196 (9)	−0.0034 (9)	0.0058 (7)	0.0018 (8)
C4	0.0368 (11)	0.0480 (13)	0.0278 (11)	−0.0035 (9)	0.0142 (8)	−0.0039 (9)
C5	0.0309 (10)	0.0421 (12)	0.0379 (12)	0.0034 (9)	0.0140 (9)	−0.0007 (9)
C6	0.0298 (10)	0.0346 (10)	0.0322 (11)	0.0052 (8)	0.0067 (8)	0.0050 (8)
C7	0.0244 (8)	0.0291 (9)	0.0219 (9)	−0.0009 (7)	0.0004 (6)	0.0005 (7)
C8	0.0257 (9)	0.0294 (9)	0.0256 (9)	−0.0003 (7)	−0.0006 (7)	0.0017 (7)
C9	0.0327 (10)	0.0371 (11)	0.0269 (10)	0.0007 (8)	0.0020 (8)	0.0004 (8)
C10	0.0318 (10)	0.0400 (12)	0.0449 (13)	0.0049 (9)	0.0081 (9)	−0.0054 (10)
C11	0.0314 (11)	0.0419 (12)	0.0554 (15)	0.0082 (9)	−0.0016 (10)	0.0047 (11)
C12	0.0414 (12)	0.0523 (14)	0.0376 (13)	0.0085 (11)	−0.0036 (10)	0.0139 (11)
C13	0.0331 (11)	0.0369 (12)	0.0568 (16)	0.0028 (9)	0.0003 (10)	−0.0137 (11)
N1	0.0247 (7)	0.0278 (8)	0.0217 (8)	0.0002 (6)	0.0047 (6)	0.0015 (6)
N2	0.0246 (7)	0.0311 (8)	0.0216 (8)	0.0017 (6)	0.0016 (6)	0.0026 (6)
N3	0.0267 (8)	0.0346 (9)	0.0236 (8)	0.0046 (7)	0.0035 (6)	0.0034 (7)
N4	0.0319 (9)	0.0440 (10)	0.0304 (9)	0.0065 (8)	0.0022 (7)	0.0108 (8)
N5	0.0803 (17)	0.0623 (15)	0.0260 (10)	0.0160 (13)	−0.0157 (10)	−0.0098 (10)
Cu1	0.02419 (16)	0.0377 (2)	0.02004 (17)	0.00875 (13)	0.00685 (11)	0.00895 (13)
S1	0.0304 (2)	0.0383 (3)	0.0196 (2)	0.0038 (2)	0.00108 (17)	0.00653 (19)
S2	0.0581 (4)	0.0487 (4)	0.0380 (3)	−0.0128 (3)	0.0077 (3)	−0.0015 (3)

### Geometric parameters (Å, º)

**Table:**

C1—N2	1.313 (2)	C10—C11	1.382 (4)
C1—C2	1.452 (3)	C10—H10	0.9300
C1—S1	1.7106 (18)	C11—C12	1.384 (4)
C2—N1	1.356 (2)	C11—H11	0.9300
C2—C3	1.382 (3)	C12—N4	1.331 (3)
C2—S1	2.8397 (19)	C12—H12	0.9300
C3—C4	1.383 (3)	C13—N5	1.115 (3)
C3—H3	0.9300	C13—S2	1.644 (3)
C4—C5	1.382 (3)	N1—Cu1	2.0463 (15)
C4—H4	0.9300	N2—N3	1.370 (2)
C5—C6	1.383 (3)	N2—Cu1	2.0267 (16)
C5—H5	0.9300	N2—S1	2.5392 (16)
C6—N1	1.337 (2)	N3—S1	2.5657 (16)
C6—H6	0.9300	N4—S1	2.9062 (18)
C7—N3	1.301 (2)	N5—S2	2.759 (2)
C7—C8	1.465 (3)	Cu1—N2^i^	2.0267 (16)
C7—S1	1.7300 (19)	Cu1—N1^i^	2.0463 (15)
C8—N4	1.338 (3)	Cu1—S2^i^	2.8124 (7)
C8—C9	1.390 (3)	Cu1—S2	2.8125 (7)
C8—S1	2.774 (2)	S1—S2^ii^	4.0094 (9)
C9—C10	1.382 (3)	S2—S1^iii^	4.0094 (9)
C9—H9	0.9300		
			
N2—C1—C2	118.82 (16)	N2—N3—S1	73.37 (9)
N2—C1—S1	113.60 (14)	C12—N4—C8	116.71 (19)
C2—C1—S1	127.58 (14)	C12—N4—S1	172.31 (16)
N1—C2—C3	122.97 (18)	C8—N4—S1	70.92 (11)
N1—C2—C1	113.12 (15)	C13—N5—S2	1.19 (15)
C3—C2—C1	123.91 (18)	N2—Cu1—N2^i^	180.0
N1—C2—S1	141.62 (12)	N2—Cu1—N1^i^	99.96 (6)
C3—C2—S1	95.40 (13)	N2^i^—Cu1—N1^i^	80.04 (6)
C1—C2—S1	28.52 (8)	N2—Cu1—N1	80.04 (6)
C2—C3—C4	118.45 (19)	N2^i^—Cu1—N1	99.96 (6)
C2—C3—H3	120.8	N1^i^—Cu1—N1	180.0
C4—C3—H3	120.8	N2—Cu1—S2^i^	91.78 (5)
C5—C4—C3	119.16 (19)	N2^i^—Cu1—S2^i^	88.22 (5)
C5—C4—H4	120.4	N1^i^—Cu1—S2^i^	91.80 (5)
C3—C4—H4	120.4	N1—Cu1—S2^i^	88.20 (5)
C4—C5—C6	119.0 (2)	N2—Cu1—S2	88.22 (5)
C4—C5—H5	120.5	N2^i^—Cu1—S2	91.78 (5)
C6—C5—H5	120.5	N1^i^—Cu1—S2	88.20 (5)
N1—C6—C5	122.9 (2)	N1—Cu1—S2	91.80 (5)
N1—C6—H6	118.6	S2^i^—Cu1—S2	180.00 (2)
C5—C6—H6	118.6	C1—S1—C7	86.79 (9)
N3—C7—C8	124.76 (17)	C1—S1—N2	28.28 (7)
N3—C7—S1	114.92 (14)	C7—S1—N2	58.51 (7)
C8—C7—S1	120.27 (14)	C1—S1—N3	59.41 (7)
N4—C8—C9	123.86 (18)	C7—S1—N3	27.38 (7)
N4—C8—C7	114.43 (17)	N2—S1—N3	31.13 (5)
C9—C8—C7	121.69 (18)	C1—S1—C8	113.89 (8)
N4—C8—S1	81.95 (12)	C7—S1—C8	27.14 (7)
C9—C8—S1	154.16 (14)	N2—S1—C8	85.63 (5)
C7—C8—S1	32.59 (9)	N3—S1—C8	54.50 (5)
C10—C9—C8	118.2 (2)	C1—S1—C2	23.90 (7)
C10—C9—H9	120.9	C7—S1—C2	110.69 (7)
C8—C9—H9	120.9	N2—S1—C2	52.18 (5)
C9—C10—C11	118.7 (2)	N3—S1—C2	83.31 (5)
C9—C10—H10	120.6	C8—S1—C2	137.77 (6)
C11—C10—H10	120.6	C1—S1—N4	140.66 (7)
C10—C11—C12	118.7 (2)	C7—S1—N4	54.21 (7)
C10—C11—H11	120.6	N2—S1—N4	112.55 (5)
C12—C11—H11	120.6	N3—S1—N4	81.50 (5)
N4—C12—C11	123.8 (2)	C8—S1—N4	27.13 (5)
N4—C12—H12	118.1	C2—S1—N4	164.06 (5)
C11—C12—H12	118.1	C1—S1—S2^ii^	95.46 (7)
N5—C13—S2	178.0 (3)	C7—S1—S2^ii^	63.79 (7)
C6—N1—C2	117.53 (16)	N2—S1—S2^ii^	82.33 (4)
C6—N1—Cu1	128.20 (13)	N3—S1—S2^ii^	70.21 (4)
C2—N1—Cu1	114.18 (12)	C8—S1—S2^ii^	64.66 (4)
C1—N2—N3	113.62 (15)	C2—S1—S2^ii^	105.86 (4)
C1—N2—Cu1	113.43 (13)	N4—S1—S2^ii^	73.36 (4)
N3—N2—Cu1	132.74 (12)	C13—S2—N5	0.81 (10)
C1—N2—S1	38.12 (9)	C13—S2—Cu1	101.44 (9)
N3—N2—S1	75.50 (9)	N5—S2—Cu1	102.00 (6)
Cu1—N2—S1	151.43 (8)	C13—S2—S1^iii^	70.19 (8)
C7—N3—N2	111.07 (15)	N5—S2—S1^iii^	69.96 (5)
C7—N3—S1	37.70 (9)	Cu1—S2—S1^iii^	151.94 (2)

Symmetry codes: (i) −*x*+1, −*y*+1, −*z*+1; (ii) *x*, *y*+1, *z*; (iii) *x*, *y*−1, *z*.

### Hydrogen-bond geometry (Å, º)

**Table:**

*D*—H···*A*	*D*—H	H···*A*	*D*···*A*	*D*—H···*A*
C3—H3···N5^iv^	0.93	2.53	3.353 (3)	147
C6—H6···N3^i^	0.93	2.35	3.143 (3)	142
C4—H4···N4^v^	0.93	2.57	3.458 (3)	161

Symmetry codes: (i) −*x*+1, −*y*+1, −*z*+1; (iv) −*x*+1, *y*+1/2, −*z*+1/2; (v) −*x*+1, *y*−1/2, −*z*+1/2.
